# The clientelism trap in Solomon Islands and Papua New Guinea, and its impact on aid policy

**DOI:** 10.1002/app5.239

**Published:** 2018-04-25

**Authors:** Terence Wood

**Affiliations:** ^1^ Development Policy Centre, Crawford School of Public Policy The Australian National University Australia

**Keywords:** aid policy, clientelism, governance, Papua New Guinea, Solomon Islands

## Abstract

Clientelism is a central feature of politics in Solomon Islands and Papua New Guinea. Most voters vote in search of personalized or localized benefit, and most politicians focus on delivering benefits to their supporters at the expense of national governance. In this article, I explain how clientelism impedes development in both countries. I then describe underdevelopment's role in causing clientelism. I also explain the resulting trap: clientelism causes underdevelopment, and underdevelopment causes clientelism. Because of the trap, clientelism will shape the two countries' politics for the foreseeable future. However, the history of other countries gives cause to believe it can be overcome in the long‐run. In the second half of the paper, I explain how change may occur. I also outline implications for aid policy, looking at how clientelism constrains the impact aid can have, and explaining how donors can act to maximize their impact in a difficult environment.

## 1 INTRODUCTION

The Western Melanesian states of Solomon Islands and Papua New Guinea (PNG) are two of the Pacific's poorest countries (World Bank, 2016a). They are also the region's two worst governed countries as measured by standard governance indicators, and quality of governance in the two countries is improving very slowly (World Bank, 2016b). Averaged over the years 2000 to 2015, PNG was the largest recipient of aid from OECD Development Assistance Committee donors in the Pacific, Solomon Islands was the second largest recipient (OECD DAC, 2017). 1Country poverty is measured here using GDP per capita adjusted for purchasing power parity. Governance scores were taken by averaging across the individual World Bank governance indicators. The most recent year that OECD aid data are available for 2015.


In the last decade, international research on clientelism has flourished. Resurgent interest in clientelism has been driven in part by clientelism's relationship with poor governance (Hicken, 2011, pp. 302–303). To date, however, clientelism in Solomon Islands and PNG, and its impact on governance, has received limited attention. Three notable exceptions are Cox (2009), Haque (2012), and Kurer (2007). Duncan and Hassall (2011) provide a useful discussion of clientelism across the Pacific. This paper builds on existing work by linking clientelism and problems of governance in Solomon Islands and PNG. It defines clientelism and shows how Solomon Islands and PNG fit the definition. Following this, it outlines the consequences of clientelism, focusing on the link between clientelism and poor governance, drawing both on international work and on examples from Solomon Islands and PNG. Then the paper examines the causes of clientelism. As it does this, the paper identifies a trap of sorts: underdevelopment and poor governance are causes of clientelism; at the same time, however, they are also effects of clientelism itself. The fact that clientelism is both a cause and effect of governance and development problems in Solomon Islands and PNG means it will not easily be transitioned away from. There is hope, however, in that, historically, other countries have exchanged clientelist politics for issues‐based programmatic politics, even if this shift has not been easy. I discuss how this has occurred in other countries and what might ultimately bring it about in Solomon Islands and PNG. In the final section of the paper, I look at the ramifications of clientelism for aid policy in Solomon Islands and PNG.

## 2 CLIENTELISM

As with most social phenomena, there is no universally agreed definition of clientelism (Hicken, 2011; Stokes, 2009). Because this article is geared around engagement with recent literature from political science, the definition that I provide here is based on definitions used in recent work in political science. The two central defining features of clientelism in this work are contingent exchange and particularism. In a democracy, contingent exchange involves voters providing electoral support for candidates, and in return being provided with material benefits. Voters' support is contingent upon receiving (or expecting to receive) benefits from candidates and politicians. Benefits from candidates and politicians are contingent upon them receiving (or expecting to receive) voters' votes (Hicken, 2011, pp. 290–294; Stokes, 2009, pp. 648–649). Voters voting in expectation of material benefits is not enough, on its own, however, to make for clientelism. Particularism is also necessary (Hicken, 2011; Stokes, 2009). Clientelist politics involves exchanges in which the benefits provided to voters are largely excludable. Instead of a policy that benefits a party's support base but that may also help non‐supporters, or the delivery of funds to an entire electoral district, which many people benefit from, including those who did not vote for the politician, the benefits of particularistic exchange are restricted: usually limited to the voter, their family, or a similar unit.

Clientelism usually comes with vote buying—the direct procurement of votes with cash in advance (Stokes, Dunning, Nazareno, & Brusco, 2013). This is an understandable pairing, although Hicken (2011, p. 296) notes that vote buying only meets the contingent exchange criterion if the politician dispensing the money is doing so believing that this will win individual voters' support in exchange for cash, rather than simply lavishing money about to prove they are a competitive candidate. Clientelism often involves candidates using interlocutors, termed “brokers” in the literature who are typically tasked with dispensing resources, winning votes, and ensuring voters make good on their part of the electoral bargain (Aspinall, 2013; Stokes et al., 2013).

## 3 CLIENTELISM IN SOLOMON ISLANDS AND PNG

Some scholars (most notably Steeves, 1996) have argued that politics in Western Melanesian countries is too fluid to accurately be described as clientelist. However, although fluidity in the form of incumbent turnover rates in Solomon Islands and PNG may be higher than in some other clientelist polities, the difference is only one of degree. As Hicken notes (Hicken, 2011, p. 303), globally, clientelist polities have higher electoral volatility on average than their programmatic counterparts. In line with this fact, electoral stability is not an element in modern definitions of clientelist politics.

The two central aspects of the definition of clientelism provided above are present in Solomon Islands and PNG. Voters have a clear preference for particularistic, localized benefits over national benefits. This can be seen in survey data, which either show voters prioritizing the local over concern with national issues (Saffu, 1989; Wood, 2013) or which show the prevalence of associated phenomena such as vote buying (Haley & Zubrinich, 2013). The particularistic nature of electoral politics in both countries is also clear in interview and case study research (Cox, 2009; Cox, 2015; Craig & Porter, 2013; Haque, 2012; Morgan & McLeod, 2006; Standish, 2007; Wood, 2014 ; Wood, 2016). Contingent exchange is also present. To an extent, pre‐election vote buying is used to signal that a candidate is competitive; however, vote buying is also used to signal that candidates are the sort of people willing to help should they be elected, and when money is spent, it is done in the anticipation that voters will repay the favour. Moreover, voters assess candidates across a range of criteria in addition to the provision of money pre‐election. Extra‐familial ties and candidates' long‐term track records of helpful behaviour are also taken account when voters decide who to vote for. This is done because voters are looking for candidates who will help them personally in an ongoing way if they are elected (Wood, 2014; Wood, 2016). Voters expect members of parliament who they vote for to help them post‐election (Morgan, 2005; also see voter and MP interviews in Wood, 2014, pp. 159–160). And candidates have at times exacted revenge on voters who they gave money to who did not subsequently vote for them (Cox, 2009). Reflecting these facts, the Varieties of Democracy dataset scores Solomon Islands in the top quintile of all countries for the clientelism of its politics, and PNG in the top decile of all countries (Coppedge et al., 2017). 2The Varieties of Democracy project is coordinated by the University of Gothenburg and the University of Notre Dame. Data are based on the assessments of expert country assessors. Globally, more than 2,800 country assessors participate in the project. The full dataset covers many different political indicators. Data used here are from version 7.1 of the dataset. They come from Section 8.7 “Particularistic or public goods.” The values I have used are countries' scores averaged across the last 15 years. If I use the most recent year's data instead of multi‐year averages, Solomon Islands' and Papua New Guinea's rankings remain similar. Data and full details can be accessed at https://www.v-dem.net/ Other features associated with clientelism, such as the use of brokers by candidates, can also be found in Solomon Islands and PNG (Filer, 1996; Wood, 2014).

## 4 THE COSTS OF CLIENTELISM

**Figure 1:**
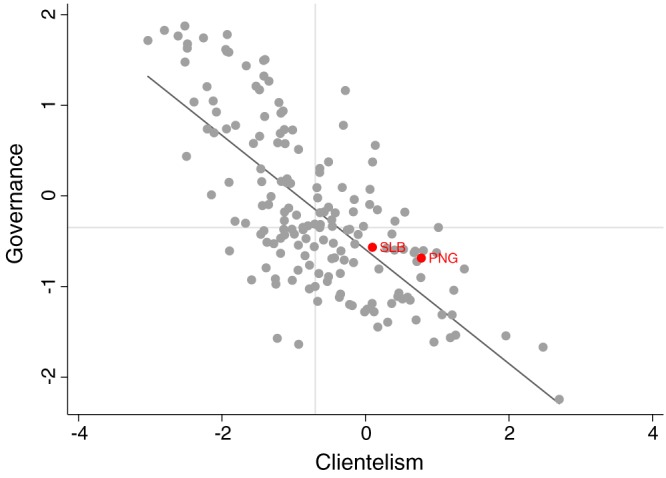
The relationship between clientelism and quality of governance. SLB = Solomon Island; PNG = Papua New Guinea

Figure 1 is a scatter plot showing the cross‐country relationship between clientelism as measured in the Varieties of Democracy dataset (Coppedge et al., 2017) and quality of governance captured as an average across all of the five different World Bank quality of governance measures (World Bank, 2016b). (See footnote 3 for more details on the Varieties of Democracy data.) Each point on the chart is a country. Solomon Islands and PNG are identified with three letter country codes. The horizontal line in the chart plots the median governance score (a number between the scores of São Tomé and Príncipe and Bosnia‐Herzegovina). The vertical line on the chart plots the median clientelism score (a number between Morocco and Iran). The diagonal line is the line of best fit. The chart shows that Solomon Islands and PNG are both worse governed than most countries and more clientelist than most countries. The two countries' close proximity to the line of best fit illustrates that they are not unusual in being both clientelist and poorly governed. The figure also demonstrates the clear relationship between clientelism and poor governance. The Adjusted *R* ^2^ from a simple bivariate regression between governance and clientelism is 0.52.

A simple bivariate relationship is not definitive evidence that clientelism causes poor governance. The relationship could be a product of a third factor, such as poverty, causing both clientelism and poor governance. Table 1 provides some evidence that this is not likely to be the case. The table shows the results of a regression in which additional control variables are added. These are all obvious confounders: the natural log of GDP per capita, the natural log of population, and a measure of democracy that draws on Freedom House and Polity IV data. All data are taken from the Varieties of Democracy dataset. Because traits such as clientelism and quality of governance are known to be slow moving, all variables are 15‐year averages.

**Table 1:** OLS regressions, correlates of World Bank governance

	Model 1	Model 2
Clientelism	−0.63*** (0.05)	−0.30*** (0.04)
Democracy		0.11*** (0.01)
GDP/capita (ln)		0.38***(0.03)
Population (ln)		−0.04** (0.02)
Constant	−0.59*** (0.06)	−3.70*** (0.37)
*N*	170	158
*R* ^2^	0.52	0.85

*Note*. Standard errors in parentheses.

* *p* < .10,

** *p* < .05,

*** *p* < 0.01.

The negative bivariate relationship between clientelism and governance that is visible in the scatter plot above can be seen in Model 1. The substantive magnitude of the relationship falls once controls are added in Model 2; however, it remains both statistically significant and substantively meaningful. This finding reduces the chance that the relationship shown in the scatterplot is a product of some obvious third factor. It does not, however, provide evidence of the direction of causality between the two variables of interest. It is possible that clientelism causes poor governance, but it could be that poor governance causes clientelism. Findings from the international literature suggest, however, that the relationship is partially driven by clientelism causing poor governance (Keefer, 2002; Keefer, 2009; Lindberg, 2010; Robinson & Verdier, 2013). Hicken (2011, p. 302) sums up the state of academic work in this area by stating that

The consensus in the literature is that clientelism has profound negative implications for the way in which democracy functions, citizen attitudes about the quality of their democracy, and the capacity of governments to produce needed public policies.

The Solomon Islands and PNG cases provide a clear illustration of clientelism causing poor governance. In both countries, voters choose who to vote for on the basis of who they think is most likely to provide them with direct localized or personalized assistance. This has an impact on governance because it selects and incentivises members of parliament to focus on delivering goods or similar assistance to their supporters, rather than concentrating on legislation or bureaucratic performance or national policy (Ketan, 2007; Kurer, 2007; Okole, 2002). As current Solomon Islands Prime Minister Ric Hou (2012, p. 1) describes it:

MPs come under considerable pressure to deliver services within their constituencies. This can mean that more time and resources are spent responding to those needs ahead of the national interest.

Voter expectations and the incentives they create are not the only causes of poor governance in Solomon Islands and PNG, but having MPs and Ministers influenced by electoral incentives to assist their supporters directly contributes in a significant way (Horscroft et al., 2017; Reilly, 2004). MPs often give out government contracts on the basis of ties rather than competitive tender (e.g., Osifelo & Dawea, 2015). Civil servants are frequently appointed on the basis of political connections (Aatai John, 2017; Fraenkel, 2005; Namosuaia, 2016; Solomon Islands Broadcasting Corporation, 2015). And MPs, who have little to fear by means of political sanction for failing on the national stage, often pay little attention to national legislation or national issues. Similarly, ministers often pay little attention to how the departments they are in charge of are functioning (Haque, 2012; Hou, 2012). Despite fiscal constraints in both countries, MPs have increased funding for constituency funds: pools of money that they are able, in effect, to spend as they will within their constituencies (Batley, 2015; Fox, 2017; Hameiri, Hughes, & Scarpello, 2017). Such funds are a direct response to electoral incentives (Batley, 2015; Hameiri et al., 2017), and although at times they do provide local benefits, they are not an efficient means of delivering public services.

## 5 THE CAUSES OF CLIENTELISM

Scholars have often attributed the particularistic nature of politics in Solomon Islands and PNG to voters holding the same expectations of politicians that their ancestors held of “big men”—community leaders, who in much of Melanesia were expected to provide material benefits to followers (for examples and discussion, see Harding, 1965; Harris, 2007; Morgan, 2005). The similarities between what was expected of big men and what is expected of modern politicians are clear. However, the idea that modern attitudes to electoral politics are a causal product of previous norms of community governance is contested. Haque (2012) has demonstrated that voters' choices in Solomon Islands and PNG can be readily explained through models of rational actors without needing to appeal to cultural legacies. Similarly, Wood (2016) argues that big men explanations of politics in Solomon Islands and PNG struggle to explain why clientelism in these countries is almost identical to clientelism in countries that have no recent history of big men governing communities. He also offers examples of voter behaviour (such as voters not voting for actual community leaders and voters voting for foreign nationals) that do not fit with a model in which voters' choices are driven by their inability to distinguish modern politicians from traditional community leaders. Culture plays a role in electoral politics in Solomon Islands and PNG (see Wood, 2016, for a full discussion of this), but the idea that clientelism is caused by voters whose expectations are trapped in the past is not particularly persuasive. For this reason, it is worth looking for broader explanations.

The main findings from existing international work are that low levels of national economic development are conducive to clientelist politics (Kitschelt & Kselman, 2013) and that, because their immediate needs are more acute, poorer people are more prone to vote in search of localized or personalized benefits (Stokes et al., 2013). The presence of governments that are unable to provide key public goods and services also contributes to clientelism (Hicken, 2011).

Solomon Islands and PNG provide clear examples of why voters vote in search of private or localized benefits in poorly governed states. Because of these countries' governance and service delivery issues, voters have not been able to rely on the government to adequately meet their basic needs. What is more, voters have never experienced elections changing these matters via policy mechanisms, or through better government performance. Under these circumstances, it makes considerable sense to vote in search of direct particularistic benefits (Haque, 2012).

A further cause of the clientelism experienced in Solomon Islands and PNG is the fact that voters face a collective action dilemma born of the nature of their countries' weak political parties and the absence of national political movements. In Solomon Islands, voters only vote for one member of parliament—their constituency's representative—out of 50. In PNG, voters vote for their constituency's representative plus a provincial representative who also sits in the national parliament—two MPs out of 111. One or two MPs can achieve very little by the way of national change on their own. In programmatic democracies, political parties based around shared interests or beliefs overcome this problem and allow voters the luxury of casting their vote knowing their MP will work alongside others to bring national‐level change. However, because Solomon Islands and PNG lack cohesive national political parties, bound by anything other than the weak ties of personal allegiance and opportunism, voters have no reason to believe their votes can be translated into national change (Standish, 2007; Wood, 2014). Without confidence that other voters in other parts of the country share their vision for an improved nation, and that reforming MPs are actually inclined to cooperate, the rational course of action for a voter, be they self‐interested or be they inclined to help as many people as possible, is to vote for someone who will help them, their family, or their community (Kurer, 2007).

## 6 THE CLIENTELISM TRAP AND ITS ESCAPE

Both the international literature and the specific cases of Solomon Islands and PNG provide good evidence that clientelism causes poor governance. Governance problems are a major cause of poverty and underdevelopment (Acemoglu, Johnson, & Robinson, 2005). Poor governance and poverty cause clientelist politics. Taken together, these facts point to a trap, one in which clientelism is preventing countries from developing, whereas at the same time, countries' underdevelopment is contributing to the clientelist politics that they suffer from. Each issue is perpetuating the other, preventing progress in either. The existence of a trap of this nature fits well with international evidence of path dependency in the quality of countries' political governance (Keefer, 2009). It also fits well with the observed ongoing development problems in Solomon Islands and PNG, and the absence of significant improvements in the quality of governance of the two countries (World Bank, 2016a; World Bank, 2016b).

The trap‐like nature of the relationship between underdevelopment and clientelist politics as experienced in Solomon Islands and PNG suggests a pessimistic prognosis for the two countries' development trajectories. However, countries—including most of today's wealthier countries—have escaped from similar situations in the past. Transitions have not been quick, nor in some cases have they been complete; however, they do provide some evidence that Solomon Islands and PNG need not stay trapped in their current state indefinitely.

A small body of work has focused on explaining cases of countries that have escaped clientelist politics. There is little evidence that legislating for stronger political parties, a solution that has often been suggested in Solomon Islands and PNG, will rid the countries of their clientelist politics. Although the clientelism of Solomon Islands and PNG is coupled with weak political parties, and although the absence of political parties is part of the collective action problem associated with the two countries' clientelism, clientelism exists in other countries where political parties are more cohesive. Indeed, parties are often an integral part of the machinery of clientelism (Hicken, 2011; Stokes et al., 2013). Functioning parties may be a necessary condition for surmounting the collective‐action problems that contribute to the clientelism experienced in Solomon Islands and PNG. However, international evidence shows that functioning political parties are not a sufficient condition for transcending clientelist politics. The nature of the parties and their objectives are crucial.

A number of political explanations have been offered for countries' transitions away from clientelist politics. Some transitions have been spurred by external threats, particularly war (Fukuyama, 2014; Shefter, 1977). However, most clientelist countries—including Solomon Islands and PNG—do not face external threats of this sort. For this reason, knowledge of this type of transformation has little practical value. Other political explanations have been offered, however, in which the key driving force is internal political change. Most of these hinge on new political movements that are either unable to succeed as clientelist machines, or which have no inclination to, owing to their desire to bring transformative change (Fukuyama, 2014; Kitschelt, 2000; Shefter, 1977). In some countries, such movements have been driven by the rise of issue‐oriented civil society engaging in systematic collective action (Abers, 1998).

This avenue for change appears possible in Solomon Islands and PNG. A politically engaged, reform‐minded middle class has grown in PNG's larger cities, particularly Port Moresby (Cox, 2014), often engaging through something akin to social movements. Similarly, new, internet‐based social movements have played an increasingly political role in Solomon Islands (Wood, 2015a). The political engagement these groups undertake is programmatic and not focused on transactional demands. It is possible that such movements could eventually grow in size, and become new political entities that help voters overcome collective action problems, and which also motivate voters into experimenting with a different type of politics. Such a transition is not guaranteed: its analogues in other countries often occurred alongside urbanization and industrial transformation. Moreover, many potential barriers stand in the way in Solomon Islands and PNG: effective collective action is inherently hard; new groups could be co‐opted by existing political operatives; groups may fail to gain much traction beyond affluent and educated urban residents. Nevertheless, this route is plausible. New groups are growing and have a clear interest in programmatic politics.

## 7 CLIENTELISM AND AID

The history of other countries provides some cause to believe that the clientelist trap facing Solomon Islands and PNG can be escaped. However, there is no reason to anticipate that such change will come rapidly. In the meantime, aid agencies need to calibrate their work to the political economy of the countries as they currently are.

It seems reasonable to anticipate that aid will be more difficult to deliver in clientelist polities. The findings of cross‐country studies fit with this belief, providing evidence that aid is less effective in countries with clientelist politics (Cruz & Keefer, 2015; Wright, 2010). However, although these studies provide evidence that aid is less likely to work in clientelist polities, their findings are relative—“less likely” is not the same as “never works”—and they speak of average relationships—the typical aid project, rather than all aid work. The findings do not mean aid is guaranteed to fail in Solomon Islands and PNG.

Although existing international work provides evidence that aid is harder to deliver successfully in clientelist countries, such work fails to identify the processes and mechanisms through which clientelism renders aid less effective. However, Solomon Islands and PNG demonstrate a number of mechanisms. The first of these is through the poor governance that clientelism brings and its impact on aid agencies' ability to spend money efficiently on services when services are delivered through government departments. In Solomon Islands and PNG, donors have used aid to improve the functioning of government departments at the same time as they have funded service delivery. In the short run, at times, this has helped. Yet, once donor engagement is scaled back, these gains have frequently been eroded by the incentives emerging from the countries' clientelist politics—ministers who have no interest in departmental functioning, or who actively subvert functioning by placing supporters in jobs, persistently undermine performance in a manner that is not easily offset by short term interventions (Haque, 2012; Wood, 2015b).

Another important challenge that emerges from the clientelism of the politics of Solomon Islands and PNG is that politicians have little political incentive to focus on maintaining infrastructure that has a public good type nature such as roads (Dornan, 2016). Solomon Islands and PNG want for functioning infrastructure in many places, and there are considerable development dividends to be had from spending aid on improving infrastructure (Dornan, 2013). However, the value of infrastructure wanes rapidly in the tropical climates of Solomon Islands and PNG unless it is maintained. If domestic political actors cannot be motivated to fund this maintenance, the development benefits of aid spent in this area are likely to be short‐lived. As a consequence, there is a risk that aid, which in better governed states could deliver sustained social and economic benefits through funding infrastructure, ends up limited to delivering short‐term benefits when focused on this work.

The nature of the countries' politics also works against delivering aid in accordance with generally recognized principals of good aid practice. In particular, the Paris Declaration and Accra Agenda for Action, alongside the Busan Partnership for Effective Development Co‐operation, are agreements in which aid donors and recipient countries have formulated a framework for best practice involving partnership based on recipient priorities (OECD, 2008; OECD, 2011). Partnership and recipient country ownership are good principals, and in many circumstances may lead to better aid. However, in practice, the recipient country partner or owner in question is not the country itself, but its government, and—to be more specific—its political leaders. If the government is poorly functioning, as is the case in Solomon Islands and PNG—a fact that is partially a product of clientelism—effective partnership and country ownership becomes difficult. (For a detailed discussion of the challenges of partnership in Solomon Islands, see Barbara (2014).) In clientelist polities, politicians have no political incentive to prioritize the welfare of their country as a whole. As a consequence, they are not ideal partners and cannot necessarily be entrusted with ownership of aid funding in the same way that politicians can be in better functioning states. This makes for a difficult partnership (Booth, 2012).

For these reasons, giving aid well in the clientelist contexts of Solomon Islands and PNG is not easy. And yet, although aid has not been able to transform either country, it has brought improvements in important areas, at least compared with a counterfactual of no aid (e.g., see Burkot & Gilbert, 2017; Wood, 2015b). Moreover, the need for the type of assistance well‐delivered aid can bring is high in both countries. The challenge for donors is to optimize the way they work in these country contexts. It is beyond the scope of this paper to provide detailed advice on how donors should do this—often best practice will vary on a project by project basis. However, some broad guidelines can be put forwards.

Donors' work needs to be based on a proper understanding of the structural drivers of governance issues. In the past, much aid to the region has simply treated governance problems as a product of capacity constraints, which it has sought to remedy through scholarships, training in government departments, and technical assistance—placing donor country personnel into government departments (Hameiri et al., 2017). It is true that governance specialists in some donor agencies often possess more sophisticated understandings of the structural drivers of governance issues (e.g., Department of Foreign Affairs and Trade, 2015; Horscroft et al., 2017). Yet, much aid practice in Solomon Islands and PNG continues to be oriented towards capacity building (e.g., see Cox, Duituturaga, & Scheye, 2012; Henderson & Boneo, 2013). This mirrors a broader phenomenon amongst other international development agencies in which sophisticated thinking on governance often fails to be reflected in aid practice (Yanguas & Hulme, 2015). Part of the reason for this is because, as I have demonstrated with my discussion of the clientelism trap, structural political issues are not easily resolved. However, even when governance issues cannot be fixed by aid donors, a rich understanding of the causes of governance problems can meaningfully improve programming. In the case of aid aimed at improving governance in clientelist polities, training should only be used when capacity constraints, rather than incentives from the political sphere, can clearly be shown to be an issue. Technical assistance may be more useful, but often not because of skillsets that foreign advisors can impart. Rather, external advisers providing technical assistance can play another role: serving as a check against maleficence emerging from the political realm—something they are well placed to do, given that, as outsiders, they are safer from to political pressure. Of course, they can only do this as long as they are engaged, but there is no reason why such engagement cannot be long‐term.

More broadly, donors need to realistically appraise what aid can achieve in the difficult contexts of Solomon Islands and PNG. In such deeply dysfunctional political environments, donors cannot jumpstart development. Aid can, however, improve human welfare in the absence of development—which is a worthwhile outcome on its own. And ongoing aid can help hold institutions together so that the countries of Solomon Islands and PNG are afforded space to develop over time. For example, donors cannot expect to build roads in Solomon Islands and PNG and then see those roads maintained by local politicians, but they can themselves fund maintenance—facilitating the economic benefits that roads bring. Similarly, donors cannot construct well‐functioning ministries of health but they can still fund important initiatives to reduce the suffering caused by ill health (e.g., Burkot & Gilbert, 2017). Likewise, donors cannot build electoral commissions that can run elections in the challenging contexts of Solomon Islands and PNG in an ongoing manner once donors have ceased funding them, but if donors are willing to invest in an ongoing way, they can help serve as a countervailing force to political dynamics that would otherwise prevent electoral commissions from being able to run adequate elections. In doing so, donors help preserve democracy, and the ongoing space for democratic development over the medium to long run (Wood, 2015b). One risk of donors becoming ongoing features of the development landscape in this way is a form of moral hazard, in which donors' ability to provide services reduces domestic pressure on governments to do this themselves. This is a risk and needs to be watched for. If donors do succeed in reducing need, and if the consequence is that voters and civil society display decreased inclination to engage productively in the political process, engagement ought to be rethought. This risk is only a possibility, however, not a certainty. As I discussed above, clientelism is partially a product of unmet need, because of this, it is equally plausible that as need falls, programmatic political engagement will actually increase bringing improved governance.

Another unavoidable challenge is that although some of the engagement I have suggested will bring tangible benefits in the short run, some gains (e.g., preventing elections from getting worse) will be harder to demonstrate, and the real benefits will only accrue over long timeframes. This brings the risk of political fatigue in donor countries. This is unavoidable, although the risk may be mitigated to an extent through the careful education of domestic political actors.

Donors may also be able to assist reforming social movements to change the dynamics of the two countries' political economies. This, however, is an area where donors should move carefully. Intervening in other countries' politics is fraught. What is more, it is possible that, although some donor funding can provide valuable assistance, too much funding may adversely affect the dynamics of reforming organizations. Also, potential movements of change, and the actors who drive them, are often hard for donors to fully understand. This will make it difficult for donors to pick potential champions within social movements. Despite these challenges, there is some scope for assistance in this area. Aiding reformers in meeting similar actors from other countries might, for example, help with the exchange of ideas. Information is also potentially useful for reformers—donors may be able to help indirectly by being transparent and encouraging government transparency as they do this. Donors can also continue to fund civil society more broadly. If nothing else, civil society organizations will often be more efficient deliverers of services than the government, at least in the short term. Careful engagement with civil society may bring benefits. However, donors should not assume that just because civil society is a necessary ingredient for political transformation, they can hasten the process of transformation simply by aiding civil society.

## 8 CONCLUSION

In this paper, I have described the clientelist nature of politics in Solomon Islands and PNG. Drawing on international work and examples from the two countries, I have explained how clientelism leads to poor governance. I have also explained how, directly and indirectly, poor governance and associated development problems contribute to clientelism. The fact that clientelism is both a cause and effect of underdevelopment points to a trap. It is unlikely that Solomon Islands and PNG will replace their clientelist politics with something more conducive to development in the short term. And yet, the rise of new social movements in both countries provides some cause for believing that change may come. The challenge for aid policy makers is to understand how best to engage in the meantime. I have argued that delivering aid that as effectively as possible in the current political environment in Solomon Islands and PNG involves understanding the political nature of governance problems in the two countries, and understanding that in many areas, the benefits of aid will not be self‐sustaining once donor engagement has ceased. If donors calibrate aid around these facts, aid can help until Solomon Islands and PNG escape their clientelism traps.
